# Analysis of Energy Dissipation Ratio in Commercial Bovine Pericardial Patches Treated with Glutaraldehyde Solution

**DOI:** 10.3390/jfb17080362

**Published:** 2026-07-28

**Authors:** Abdulrahman Alblowi, Siyu Lin, Olivier Bouchot, Jeremy Lagrange, Nicla Settembre, Alain Lalande, Serguei Malikov

**Affiliations:** 1 DCAC, INSERM, Université de Lorraine, 54000 Nancy, France; 2 Department of Vascular and Endovascular Surgery, Nancy Regional University Hospital, University of Lorraine, 54500 Vandoeuvre-les-Nancy, France; 3 ICMUB CNRS UMR 6302, Université Bourgogne Europe, 21000 Dijon, France; siyu.lin@u-bourgogne.fr; 4 Division of Vascular and Endovascular Surgery, Department of General Surgery, King Fahad Hospital of the University, College of Medicine, Imam Abdulrahman Bin Faisal University, Dammam 34211, Saudi Arabia; 5 Département de Chirurgie Cardiovasculaire et Thoracique, CHU Dijon Bourgogne, ICMUB UMR 6302, CNRS, Université Bourgogne Europe, 21000 Dijon, France; 6 IHU Infiny, CHRU Nancy, 54500 Vandoeuvre-les-Nancy, France; 7 Pôle Imagerie, CHU Dijon Bourgogne, ICMUB UMR 6302, CNRS, Université Bourgogne Europe, 21000 Dijon, France

**Keywords:** bovine pericardial patches, xenograft material, aortic graft, biomechanics, stress–strain behavior, fatigue test

## Abstract

**Background**: Energy dissipation reflects the viscoelastic behavior of biological tissues and plays a key role in arterial elastic recoil and diastolic flow support. In the native aorta, efficient storage and release of mechanical energy are essential for maintaining ventriculo–aortic coupling. The energy dissipation ratio (EDR) quantifies the proportion of mechanical energy lost during a loading–unloading cycle and may provide insight into the biomechanical performance of aortic substitutes. Bovine pericardial patches (BPPs) are widely used in cardiovascular surgery for arterial reconstruction, patch angioplasty, and tubular replacement. Today, EDR has not been systematically investigated in BPPs. **Methods:** Forty glutaraldehyde-treated BPPs from four commercial manufacturers (*n* = 10 per supplier) were subjected to low-cycle fatigue testing using a uniaxial tensile system under controlled physiological conditions (37 °C). Standardized bone-shaped specimens were tested at progressive strain percentage levels. Thickness, EDR, and the percentage of specimens failing to reach progressively higher strain levels were evaluated from stress–strain hysteresis loops. **Results:** BPPs thickness ranged from 0.253 to 0.608 mm, with no significant differences among most groups. For the 10% strain, all BPPs reached the target deformation and demonstrated comparable EDR values. In detail, the mean EDR was 21.40 ± 7.02% for Edwards Lifesciences, 24.95 ± 6.80% for Supple Peri-Guard (Baxter), 24.99 ± 6.04% for Xenosure (LeMaitre), and 23.37 ± 6.12% for Invengenx–Tisgenx, with no statistically significant intergroup differences (*p* > 0.05). For the 20% strain, only 18.75% of specimens remained structurally intact, and variability increased. At 30% strain, structural failure occurred in nearly all samples. No significant orientation-dependent differences were observed. **Conclusions:** Commercially available BPPs exhibit similar biomechanical behavior under moderate deformation. However, tolerance to higher strain is limited. EDR analysis provides a clinically relevant parameter to assess elastic performance and may contribute to optimizing aortic substitute selection.

## 1. Introduction

The loss of energy test, commonly and historically applied to solid materials, measures the energy absorbed by a material prior to fracture, serving as a critical indicator of its toughness and resistance to mechanical failure [1]. In elastic materials, deformation occurs in response to an applied force. These materials are capable of returning to their original shape once the load is removed, reflecting their characteristic reversible mechanical behavior [2]. For the arterial tissue, the presence of elastin and collagen within the vascular wall imparts elasticity, allowing it to deform in response to pressure and return to its original shape upon unloading [3]. For a normal artery, approximately 20% of mechanical energy is dissipated during each loading-unloading cycle due to the viscoelastic nature of arterial walls, while up to 80% is temporarily stored as elastic energy within the vessel wall [3]. This stored energy is subsequently released, facilitating arterial recoil and contributing to blood propulsion during the diastolic phase. This phenomenon is known as the Windkessel effect [3,4,5].

Bovine pericardial patches (BPPs) are used in cardiovascular surgery, particularly in aortic procedures, as either patches or tubular conduits [6,7]. Their popularity stems from consistent reliability, ease of use, long-term durability, and mechanical strength. Additional benefits include reduced suture line bleeding, off-the-shelf availability, resistance to aneurysmal degeneration, and a lower risk of postoperative stenosis and infection [8]. In aortic surgery, the use of replacement materials with biomechanical properties closely resembling those of the native aorta may reduce the risk of postoperative complications such as cardiac hypertrophy and hypertension [9,10].

The degree of aortic stretching during the cardiac cycle varies according to anatomical location and corresponding physiological function. The ascending aorta demonstrates the highest distensibility and circumferential strain [11]. This distensibility progressively decreases along the aorta, with significantly lower values observed in the abdominal segment. Reported circumferential strain values range from approximately 29% in the proximal aorta to around 5% in the abdominal aorta [12,13,14]. This value is reflected by the quantity of elastin and collagen in the aortic wall. In the ascending aorta, elastin constitutes approximately 27% of the arterial wall, while collagen accounts for about 22%. In contrast, in the distal part of the aorta, the elastin content decreases to around 17%, whereas collagen increases markedly to approximately 53% [15].

Greater energy loss reflects reduced efficiency of the arterial wall in recovering stored mechanical energy, indicating that more energy is dissipated within the tissue during each cycle. The energy dissipation ratio (EDR) is defined as the normalized fraction of total mechanical energy input that is lost during a single loading–unloading cycle [16]. In arterial mechanics, a higher EDR denotes increased internal friction and reduced elastic efficiency, indicating altered wall behavior due to structural remodeling, such as elevated collagen content, elastin fragmentation, or smooth muscle disorganization. Conversely, a lower EDR reflects more efficient elastic recoil and a structurally intact, healthier arterial wall [17,18,19].

Evaluating the EDR in synthetic and biological aortic substitutes is crucial for assessing their capacity to store and release elastic energy and for guiding the selection and development of optimal replacement materials [20]. Despite the widespread clinical use of BPPs in aortic reconstruction, their energy-dissipation behavior has not been systematically characterized. This represents an important knowledge gap because native aortic replacement materials should ideally reproduce not only strength and durability but also viscoelastic behavior and energy-handling properties comparable to those of the native aorta. Therefore, the novelty of this study lies in the first systematic assessment of the energy-dissipation ratio of a commercial glutaraldehyde-treated bovine pericardial patch when used as a conduit for aortic tissue replacement. By analyzing the full loading–unloading stress–strain response, this study provides a new biomechanical characterization of bovine pericardial patch material and explores its suitability as an aortic substitute. To our knowledge, this specific parameter has not previously been investigated for this application.

This study aims to characterize the biomechanical performance of commercially available BPPs treated with glutaraldehyde solution when used as a conduit for aortic tissue replacement by assessing EDR across strain levels. The energy loss parameter reflects the overall stress–strain behavior of the tissue throughout the entire loading cycle, providing a more stable and reliable measure.

## 2. Methods

A total of 40 BPPs, preserved in glutaraldehyde solution, were obtained from four different companies, with ten patches collected from each supplier. The products included Edwards Lifesciences^®^ (Edwards Lifesciences Corporation, Irvine, CA, USA; model no. 4700; 10 × 15 cm), Supple Peri-Guard^®^ (Baxter International Inc., Deerfield, IL, USA; model no. PC-1016SN; 10 × 16 cm), Xenosure^®^ (LeMaitre Vascular Inc., Burlington, MA, USA; model no. 10BV16; 10 × 16 cm), and Invengenx^®^ (Tisgenx Inc., Irving, TX, USA; model no. XM-21; 7 × 10 cm). BPPs were chemically sterilized and delivered in a condition appropriate for clinical use. Based on available manufacturer labeling: Edwards Lifesciences^®^ patches are preserved in glutaraldehyde, sterilized with ethanol and polysorbate-80, and treated with sodium hydroxide (storage: 10–25 °C); Supple Peri-Guard^®^ with ethanol and propylene oxide plus sodium hydroxide (storage: 20–25 °C); Xenosure^®^ to a sterility assurance level of 10^−6^ (storage: 0–40 °C); and Invengenx^®^ using proprietary elixP™ fixation technology (storage: 0–40 °C).

Each BPP was sectioned into four bone-shaped specimens—two oriented horizontally and two vertically—yielding 40 specimens per supplier group (20 horizontal and 20 vertical), and a total of 160 specimens across all four groups. Each specimen measured 50 mm × 10 mm, comprising an effective testing length of 36 mm, while the remaining 14 mm was allocated for clamping during mechanical testing. Thickness measurements were obtained at three distinct points along the effective testing length using an electronic thickness micrometer (Litematic VL-50, Mitutoyo Corporation, Kawasaki, Japan).

Low-cycle fatigue testing was conducted using a horizontal uniaxial tensile testing system (LM1, TA Instruments, ElectroForce^®^ System Group, Eden Prairie, MN, USA) to evaluate the energy dissipation ratio of the BPPs under conditions of high strain and low-cycle counts, representative of dynamic loading and severe physiological stress. Prior to testing, each specimen underwent 10 preconditioning cycles at a displacement of 0.125 mm per motor step at 1 Hz, to stabilize the mechanical response and minimize hysteresis variability before data acquisition. All tests were performed in distilled water maintained at 37 ± 0.1 °C under temperature-controlled hydrated conditions. Distilled water was selected to ensure consistent hydration across all specimens and to avoid variability in ionic concentration between testing sessions, while maintaining tissue temperature comparable to the physiological environment.

The test assessed the fatigue performance of the materials by subjecting them to repeated deformation within the low-cycle fatigue regime, typically involving a limited number of cycles at elevated stress amplitudes. In this study, the displacement amplitude was progressively increased as follows: 1.8 mm at 0.1 Hz (1 cycle per motor), 3.6 mm at 0.1 Hz, 5.4 mm at 0.1 Hz, and 7.2 mm at 0.1 Hz, corresponding to approximately 10%, 20%, 30%, and 40% strain, respectively. Strain was calculated using *ε* = Δ*L*/*L*_0_ × 100, where *L*_0_ = 18 mm, corresponding to half the effective gauge length, as the LM1 system applies displacement symmetrically from both clamps simultaneously. Each specimen was tested until the completion of the loading protocol or until material rupture occurred during the low-cycle fatigue testing process. The amplitude step consisted of a single loading–unloading cycle per motor step. All measurements were taken within a time frame of 0.0001 s to ensure precise data. The software utilized during the experiments for the registration of the data was WinTest^®^ 8 (TA Instruments, ElectroForce^®^ System Group, USA). The data were then analyzed to determine the EDR and elastic modulus at different strain percentages. Cycles that were affected by rupture, incomplete loading, or an unexpected reduction in the energy dissipation ratio relative to the preceding cycle, indicative of micro-damage or early failure, were excluded from the analysis.

Based on the measured specimen thickness, the initial distance between the clamps of the uniaxial tensile testing system, and the applied force at various strain levels, the energy dissipation ratio was calculated across different strain percentages, according to Equation (1):

(1) $$Energy\;Loss=\;\int_{\varepsilon_{0}}^{\varepsilon_{max}}\frac{{F\;L}_{0}}{\omega \;t\;∆L}{d\varepsilon }_{loading}-\;\int_{\varepsilon_{max}}^{\varepsilon_{0}}\frac{{F\;L}_{0}}{\omega \;t\;∆L}{d\varepsilon }_{unloading}$$

Indeed, Equation (1) is the mathematical formulation used to calculate the biomechanical energy loss (hysteresis loss) during cyclic loading and unloading; where *F*: Applied force; *L*_0_: Initial length of the specimen; *w*: Width of the specimen; *t*: Thickness of the specimen; Δ*L*: Elongation under load; *ε*: Strain; *ε*_0_: Initial strain; *ε_max_*: Maximum applied strain; *ε_loading_*: Strain during loading; *ε_unloading_*: Strain during unloading phases.

**Figure 1:**
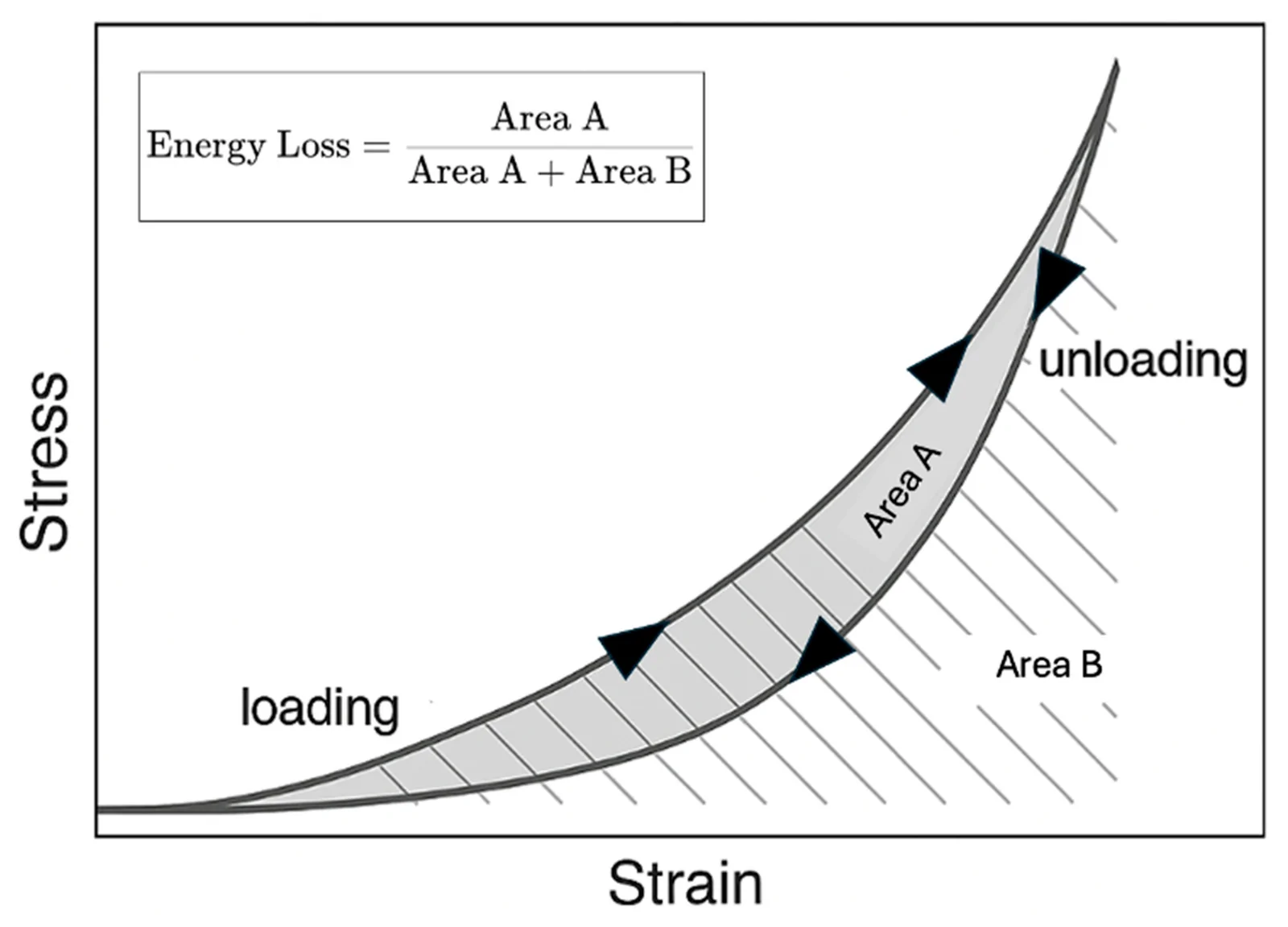
Schematic representation of the stress–strain curve showing the mechanical response of the biomaterial during cyclic loading and unloading. The loading curve shows the increase in stress as strain rises, whereas the unloading curve reflects stress reduction as the material recoils. The shaded region between the two curves forms the hysteresis loop, which represents the mechanical energy dissipated during one deformation cycle. In this diagram, Area A corresponds to the recoverable elastic energy released during unloading, while Area B represents the energy retained as deformation-related loss. The energy loss ratio is calculated as Area A/(Area A + Area B), and it quantifies the proportion of energy dissipated relative to the total mechanical energy input. A higher hysteresis area indicates greater energy dissipation and lower elastic efficiency of the biomaterial.

To evaluate the biomechanical behavior of the BPPs, the energy loss was then calculated as the percentage of strain energy dissipated between the loading and unloading curves of the stress–strain relationship. This parameter represents material hysteresis and characterizes the viscous component of the tissue, as shown in Figure 1.

The primary endpoint is the measurement of the Energy Dissipation Ratio (EDR) of the different BPPs at a predefined percentage of strain. The secondary endpoint is the evaluation of intra-sample and inter-sample variability to determine the consistency and reliability of the biomechanical performance of BPPs.

GraphPad Prism 7 software (GraphPad Software, San Diego, CA, USA) was utilized for parameter fitting and statistical analysis. Data derived from the BPPs’ mechanical tests using the tensile testing LM1 system (TA Instruments, ElectroForce^®^ System Group, USA) and measured specimen thickness were used to calculate the EDR.

Continuous variables are presented as means ± standard deviation (SD). Across the four companies, based on the two pieces sharing the same orientation for dedicated horizontal and vertical comparison analyses, the mean parameter values, including thickness and EDR, were calculated. Normality was assessed using the Shapiro–Wilk test. The four groups were compared using ANOVA with Tukey’s post hoc test for parametric data and the Kruskal–Wallis test with Dunn’s correction for non-parametric data. A *p*-value of less than 0.05 was considered statistically significant.

## 3. Results

Biomechanical testing was conducted on BPPs provided by four commercial suppliers. The mean thickness of patches from Edwards Lifesciences^®^ was 0.441 ± 0.047 mm (median, 0.439 mm; range, 0.353–0.508 mm). Supple Peri-Guard^®^ patches demonstrated a mean thickness of 0.457 ± 0.069 mm (median, 0.453 mm; range, 0.315–0.548 mm). In comparison, Xenosure^®^ patches exhibited lower thickness values, with a mean of 0.387 ± 0.075 mm (median, 0.408 mm; range, 0.253–0.465 mm). Invengenx^®^ patches were the thickest among the evaluated products, with a mean thickness of 0.484 ± 0.082 mm (median, 0.463 mm; range, 0.393–0.608 mm) (Table 1).

With the exception of a significant difference between the Xenosure^®^ and Invengenx^®^ groups (mean difference −0.097; 95% CI −0.181 to −0.013; *p* = 0.018), there were no other statistically significant differences in thickness among groups. Additionally, no significant intra-group differences in thickness were observed between vertical and horizontal directions.

**Figure 2:**
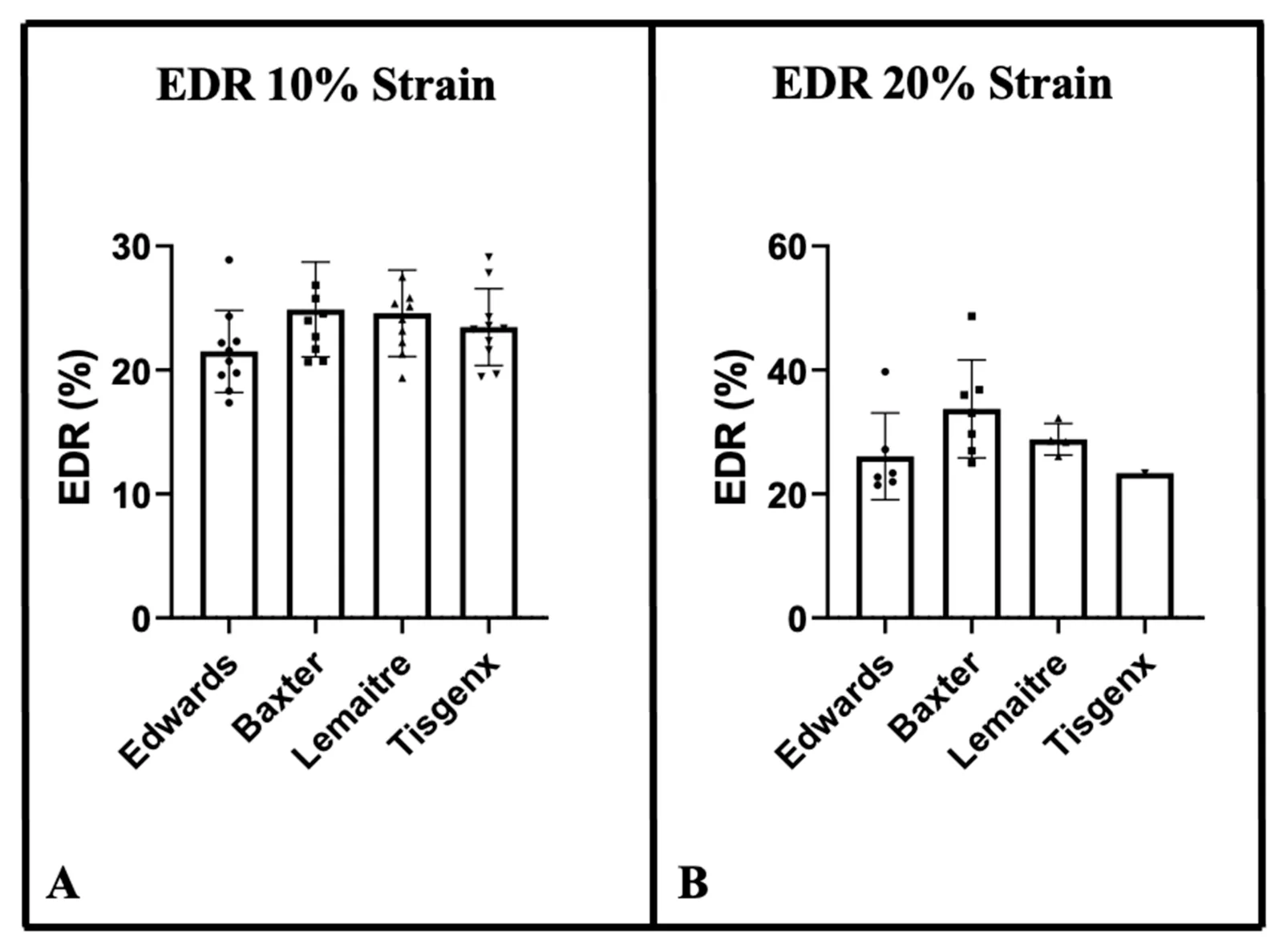
Energy dissipation ratio (EDR, %) of BPPs from four manufacturers (Edwards, Baxter, Lemaitre, and Tisgenx) measured at 10% strain (**A**) and 20% strain (**B**). Bars represent the mean EDR for each group, and the individual dots indicate the corresponding specimen-level measurements. Mean EDR value obtained from the specimens from each BPP (*n* = 10 patches per manufacturer).

For the 10% strain measurement, the mean EDR for Edwards Lifesciences^®^ patches was 21.40 ± 7.02%, with values ranging from 10.31% to 47.96%. Supple Peri-Guard^®^ patches demonstrated a mean EDR of 24.95 ± 6.80% (range: 16.48–40.65%). Xenosure^®^ patches showed a mean EDR of 24.99 ± 6.04% (range: 15.73–38.96%). Invengenx^®^ patches exhibited a mean EDR of 23.37 ± 6.12%, with values ranging from 15.68% to 45.01%. There were no statistically significant differences between the groups. These results indicate comparable energy dissipation behavior at 10% strain across all tested materials (Figure 2A).

In addition, there were no significant differences in EDR for 10% strain between vertical and horizontal orientation.

For the 20% strain measurement, the mean EDR for Edwards Lifesciences^®^ patches was 26.07 ± 6.98% (range: 21.45–39.71%; *n* = 6). Supple Peri-Guard^®^ patches demonstrated a mean EDR of 33.73 ± 7.92% (range: 25.03–48.66%; *n* = 7). The Xenosure^®^ showed a mean EDR of 28.83 ± 2.55% (range: 26.06–32.24%; *n* = 4). For Invengenx^®^ patches, only one specimen remained available at 20% strain, with an EDR value of 23.35%. No statistically significant differences were observed between the groups (Figure 2B) despite the apparent dispersion of the values.

**Figure 3:**
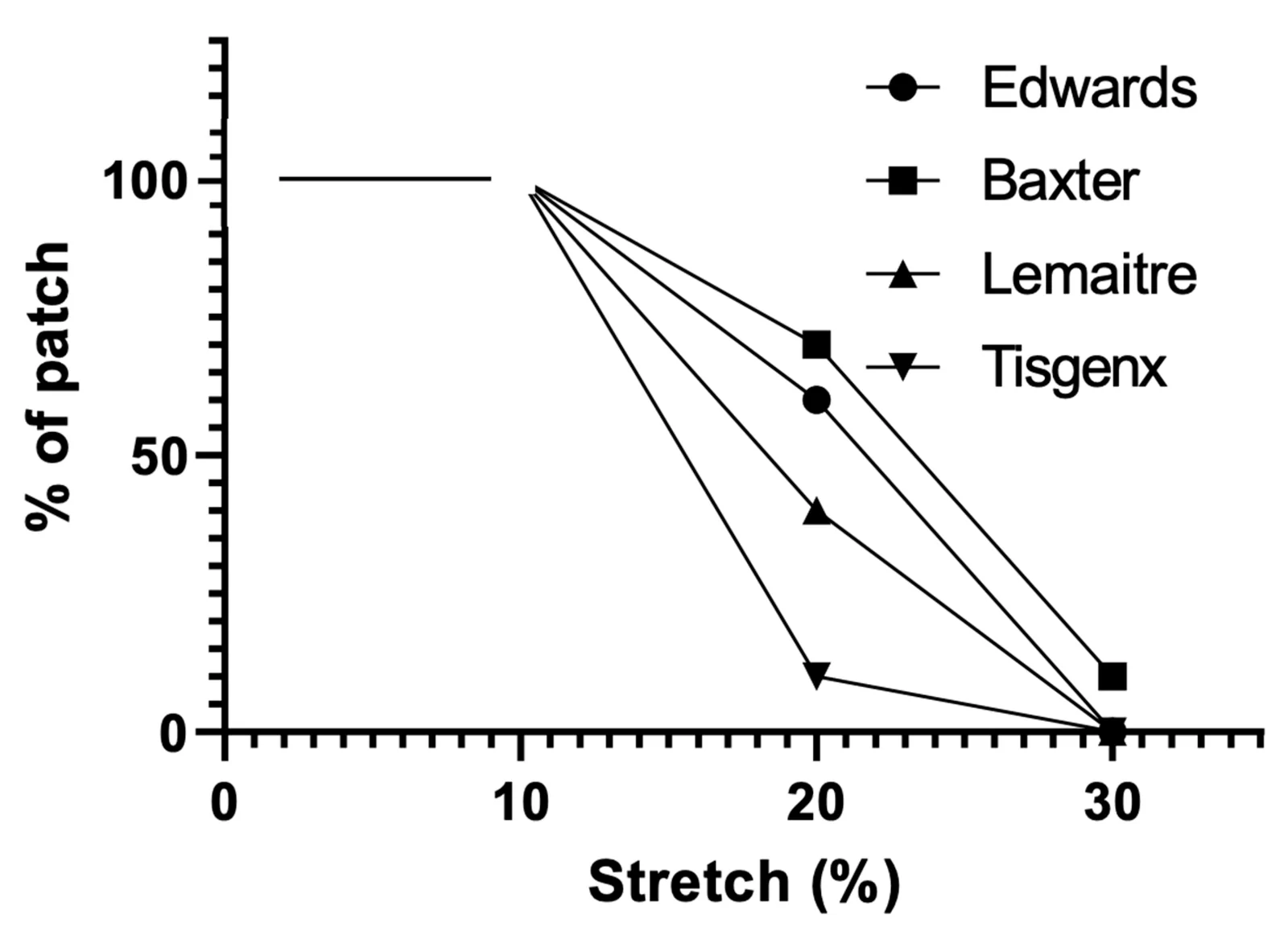
Percentage of BPPs failing to reach each predefined strain level during EDR testing. The proportion of BPPs failing to reach the target strain increased with increasing strain levels, reflecting the progressive mechanical limitations of the tested materials under cyclic loading conditions.

All BPPs from all manufacturers successfully reached 10% strain during the EDR test, allowing complete analysis at this deformation level. In contrast, 18.75% of all patches reached 20% strain for the EDR test, including 13 Edwards Lifesciences^®^ patches (32.5%), 10 Supple Peri-Guard^®^ patches (25%), 6 Xenosure^®^ patches (15%), and 1 Invengenx^®^ patch (2.5%). At 30% strain during the EDR test, only 1.25% of the total samples were able to sustain deformations. Specifically, two Supple Peri-Guard^®^ patches (5%) reached this level, whereas no BPPs from Edwards Lifesciences^®^, Xenosure^®^, or Invengenx^®^ achieved 30% strain for the EDR test (Figure 3).

## 4. Discussion

This study characterized the biomechanical properties of BPPs commonly used in cardiovascular surgery. By systematically evaluating BPPs from four commercial suppliers, we aimed to provide a clearer vision of biomechanical behavior and to elucidate how inter-manufacturer differences may influence clinical performance and outcomes.

Low-cycle fatigue testing was employed to evaluate the biomechanical behavior of different BPPs under varying strain levels and limited cycle numbers, conditions that are intended to simulate physiological hemodynamic loading and intraoperative mechanical manipulation. Within this framework, the EDR quantifies the proportion of mechanical energy lost during a loading–unloading cycle. Reduced arterial compliance and impaired buffering capacity lead to adverse mechanical consequences, increasing the risk of left ventricular hypertrophy and impairing coronary perfusion and compromises coronary perfusion [17,18,19].

In our study, the thickness of the samples varied between 0.253 and 0.608 mm. These findings are consistent with previous studies indicating that the typical thickness of pericardial tissue generally ranges between 0.4 and 1.0 mm [21,22,23]. The values obtained from the different suppliers were within the normal range.

In addition, no significant differences were observed across the different patch types regarding thickness or biomechanical evaluations, and there was no thickness-related intra-sample variability, indicating a homogenous biomaterial. The results of EDR 10% and 20% show no statistically significant differences among the groups.

The absence of significant intergroup differences in EDR across the four commercial BPPs can be hypothetically attributed to the dominant and unidirectional effect of glutaraldehyde cross-linking on the extracellular matrix of BPPs. Glutaraldehyde treatment induces covalent cross-linking of collagen fibers, reduces the mobility of fibrous network components, and substantially diminishes the contribution of elastin to the overall mechanical response [24,25]. This biochemical homogenization of the tissue architecture likely overrides any pre-existing inter-sample variability attributable to donor animal age, anatomical harvesting site, or native tissue composition. As a result, the viscoelastic behavior of the treated tissue and consequently the hysteresis loop characteristics from which EDR is derived converge toward a common range across all products.

The concept of energy loss is relatively new in the field of biomaterials and is used to assess tissue degeneration. A previous study applying this approach to ascending aortic specimens demonstrated that samples exhibiting higher energy loss also showed more pronounced medial degeneration compared with tissues of similar size but lower energy loss [26]. At present, there is no validated threshold to define material degeneration. Complementary histological assessment following repeated strain cycles would be required to characterize microscopic structural changes. Furthermore, simulating intraoperative conditions, such as clamping with a cardiovascular clamp, followed by histological reassessment, could provide valuable insight into the mechanical fragility and clinical suitability of the BPPs.

The potential relationship between the use of BPPs and subsequent cardiac remodeling, including hypertrophy, remains insufficiently explored. Mechanical properties such as stiffness, as well as graft length, configuration (patch versus tubular reconstruction), and anatomical implantation site, may influence ventriculo–aortic coupling and long-term cardiac adaptation. These factors warrant further investigation to better understand their potential impact on postoperative hemodynamics and myocardial workload.

Conventional stress–strain analysis characterizes the elastic behavior of biological tissues by describing the relationship between applied load and resulting deformation, providing parameters such as elastic modulus and ultimate tensile strength [27]. However, this approach captures only a single direction of the loading curve. In contrast, the EDR is derived from the area enclosed within the loading and unloading curves of the stress–strain relationship and quantifies the fraction of mechanical energy that is dissipated as heat and vessels rather than recovered as elastic recoil [28]. This distinction is clinically meaningful in the aortic context: the native aorta does not merely resist deformation, but actively stores and releases elastic energy during each cardiac cycle to sustain diastolic blood flow—the so-called Windkessel effect [29,30]. The EDR, therefore, provides a functionally complementary and clinically relevant parameter that conventional mechanical testing cannot capture, and its systematic assessment represents a key methodological contribution of the present study [26].

Several limitations of the present study should be acknowledged. First, although the in vitro mechanical assessment was designed to approximate clinically relevant loading conditions, it cannot fully reproduce the complex in vivo hemodynamic environment, including pulsatile flow and multidirectional mechanical loading. While uniaxial tensile testing remains the most widely accepted method for the biomechanical characterization of bovine pericardial biomaterials, it does not fully capture their multiaxial mechanical behavior; therefore, future studies incorporating biaxial testing and pulsatile flow models would provide a more comprehensive evaluation. Second, incomplete information regarding the harvesting site and age of the bovine donors may have contributed to biological variability among specimens. Finally, biomechanical testing was performed in distilled water rather than physiological saline or phosphate-buffered saline, which may not fully replicate the ionic environment encountered in vivo. These limitations should be considered when interpreting the present findings.

In future studies, MRI of the aorta may serve as a valuable noninvasive imaging modality for evaluating patients treated with BPP or tubular graft reconstruction, particularly for the assessment of long-term structural and functional outcomes using conventional MRI in combination with 4D flow MRI. Further studies integrating histological analysis with in vivo imaging assessments are warranted to better characterize long-term performance and remodeling behavior.

## 5. Conclusions

In this study, bovine pericardial patches from four commercial suppliers demonstrated comparable thickness and biomechanical behavior at 10% strain, with no significant intergroup differences in EDR. Although minor variability in thickness was observed, all values remained within the expected physiological range. At higher strain levels, a marked reduction in structural integrity was noted across materials, highlighting potential limitations under excessive mechanical stress. The EDR values observed fall within a clinically relevant biomechanical spectrum and may provide insight into elastic performance and tissue resilience. These findings support the overall mechanical consistency of currently available BPPs under moderate deformation, while emphasizing the need for further investigation under physiologic loading conditions. Future studies incorporating histological analysis will be required to define the EDR threshold degeneration level and to simulate intraoperative conditions.

## Figures and Tables

**Table 1:** Description of BPPs’ thickness (in mm) from different suppliers, calculated from 10 samples.

Group	Mean ± SD	Median	Min–Max	p25	p75	IQR
Edwards Lifesciences^®^	0.441 ± 0.047	0.439	0.353–0.508	0.409	0.478	0.069
Supple Peri-Guard^®^	0.457 ± 0.069	0.453	0.315–0.548	0.417	0.513	0.096
Xenosure^®^	0.387 ± 0.075	0.408	0.253–0.465	0.326	0.460	0.134
Invengenx^®^	0.484 ± 0.082	0.463	0.393–0.608	0.406	0.575	0.169

Mean ± SD: Mean and standard deviation. p25, p50, p75: 25th percentile, median, and 75th percentile. IQR: Interquartile range.

## Data Availability

The raw data supporting the conclusions of this article will be made available by the authors on request.
